# Variation in Antiosteoporotic Drug Prescribing and Spending Across Spain. A Population-Based Ecological Cross-Sectional Study

**DOI:** 10.3389/fphar.2018.00342

**Published:** 2018-04-13

**Authors:** Gabriel Sanfélix-Gimeno, Julián Librero-López, Gracia Modroño-Riaño, Salvador Peiró, Clara L. Rodríguez-Bernal

**Affiliations:** ^1^Fundación para el Fomento de la Investigación Sanitaria y Biomédica de la Comunitat Valenciana (FISABIO-Salud Pública), Valencia, Spain; ^2^Red de Investigación en Servicios de Salud en Enfermedades Crónicas (REDISSEC), Valencia, Spain; ^3^Centro de Investigación Biomédica del Gobierno de Navarra (Navarrabiomed), Edificio de Investigación, Pamplona, Spain; ^4^Servicio de Farmacia en Atención Primaria y Especializada, Gijón, Spain; ^5^Servicio de Salud del Principado de Asturias, Oviedo, Spain

**Keywords:** osteoporosis, geographical variation, pharmaceutical utilization, expenditure, medication, hip fracture prevention

## Abstract

**Introduction:** Evidence has shown that utilization of antiosteoporotic medications does not correspond with risk, and studies on other therapies have shown that adequacy of pharmaceutical prescribing might vary between regions. Nevertheless, very few studies have addressed the variability in osteoporotic drug consumption. We aimed to describe variations in pharmaceutical utilization and spending on osteoporotic drugs between Health Areas (HA) in Spain.

**Methods:** Population-based cross-sectional ecological study of expenditure and utilization of the five therapeutic groups marketed for osteoporosis treatment in Spain in 2009. Small area variation analysis (SAVA) methods were used. The units of analysis were the 168 HA of 13 Spanish regions, including 7.2 million women aged 50 years and older. The main outcomes were the defined daily dose (DDD) per 1000 inhabitants and day (DDD/1000/Day) dispensed according to the pharmaceutical claims reimbursed, and the expenditure on antiosteoporotics at retail price per woman ≥50 years old and per year.

**Results:** The average osteoporosis drug consumption was 116.8 DDD/1000W/Day, ranging from 78.5 to 158.7 DDD/1000W/Day between the HAs in the 5th and 95th percentiles. Seventy-five percent of the antiosteoporotics consumed was bisphosphonates, followed by raloxifene, strontium ranelate, calcitonins, and parathyroid hormones including teriparatide. Regarding variability by therapeutic groups, biphosphonates showed the lowest variation, while calcitonins and parathyroid hormones showed the highest variation. The annual expenditure on antiosteoporotics was €426.5 million, translating into an expenditure of €59.2 for each woman ≥50 years old and varying between €38.1 and €83.3 between HAs in the 5th and 95th percentiles. Biphosphonates, despite accounting for 79% of utilization, only represented 63% of total expenditure, while parathyroid hormones with only 1.6% of utilization accounted for 15% of the pharmaceutical spending.

**Conclusion:** This study highlights a marked geographical variation in the prescription of antiosteoporotics, being more pronounced in the case of costly drugs such as parathyroid hormones. The differences in rates of prescribing explained almost all of the variance in drug spending, suggesting that the difference in prescription volume between territories, and not the price of the drugs, is the main source of variation in this setting. Data on geographical variation of prescription can help guide policy proposals for targeting areas with inadequate antiosteoporotic drug use.

## Introduction

Osteoporotic fractures, and among them hip fractures – the more severe clinical manifestation of osteoporosis – account for the majority of fracture-related health care expenditure and mortality in men and women aged 50 years and over (Johnell and Kanis, 2006; Ström et al., 2011). Although directing prevention strategies at high-risk groups is a key component of cost-effective policies seeking to lower the incidence and impact of osteoporotic fractures, evidence has shown that utilization of antiosteoporotic medications does not correspond with risk, and the Spanish setting is a good example: whereas Spain is one of the European (and worldwide) countries with a lower incidence of osteoporotic fracture (Kanis et al., 2012; Hernlund et al., 2013), antiosteoporotic medications are among the most widely prescribed drugs in the Spanish National Health System (sNHS). A report from 2010 analyzing variability in the consumption of several therapeutic drugs in 15 developed countries (including the United States, Canada, and some European countries) placed Spain as the country with the highest utilization of antiosteoporotic drugs (Richards, 2010). Moreover, previous studies suggest that various countries including Spain are seeing a massive use of osteoporotic treatments in young women with a very low risk of fracture, while there is a significant underuse in women (and men) at a high risk of fracture, including those who have already suffered a major osteoporotic fracture (de Felipe et al., 2010; Sanfélix-Genovés et al., 2013b; Wang et al., 2013; Yu, 2017).

This might be due to the existence of considerable uncertainty about how to quantify the risk, who should receive densitometry testing, who should be treated, what treatment should be used, and for how long (Bolland and Grey, 2010; Sanfélix-Genovés et al., 2013a; Hurtado et al., 2014). Furthermore, and beyond the evidence provided by clinical trials and the agreements reached by expert consensus panels, treatment decisions and the choice of a particular treatment may be influenced by patient characteristics, physician, and organizational factors, pharmaceutical promotion, and healthcare system characteristics such as copayment, accessibility, or others (Eisenberg, 2002).

The lack of strong, coherent, and widely accepted guidance for pharmacological treatment aimed at preventing fragility fractures may result in a wide variation in the patterns of drug use among neighboring populations with very similar demographic and epidemiologic characteristics. However, studies examining within-country geographical variations in pharmaceutical prescribing have been scarce (Anis et al., 1996; Dubois et al., 2002) until recent years (Zhang et al., 2010, 2012; Donohue et al., 2012) and very few have addressed –and only partially – the variability in osteoporotic drug consumption (Rocha et al., 2006; Devold et al., 2010; Crilly et al., 2014; Shah et al., 2017). Knowledge of these variations will be essential to interpret the behavior of healthcare providers and to define public policies aiming to reduce both the burden of osteoporosis on patients and unnecessary expenditure on pharmacological treatment.

The aims of this study are to describe variations in pharmaceutical utilization and spending on osteoporotic drugs between Health Areas (HA) in Spain in 2009 and to analyze the observed variation using small area variation analysis (SAVA) methods.

## Materials and Methods

### Design

Population-based cross-sectional ecological study of expenditure and utilization of the five therapeutic groups marketed for osteoporosis treatment in Spain.

### Population

The units of analysis were the 168 HAs of the 13 Spanish Autonomous Communities (AC) participating in the study (Andalucia, Aragon, Asturias, Balearic Islands, Canary Islands, Castilla-Leon, Catalonia, Extremadura, Madrid, Murcia, Navarra, and the Valencia Region). These HAs, after excluding people not entitled to pharmaceutical benefits, covered a population of about 39.8 million inhabitants in 2009 (roughly 80% of the Spanish population at that time), of which 7.2 million were women aged 50 years and over.

### Setting

The sNHS – during the study period, previous to the 2012 healthcare reforms on entitlement and copayment – provided universal healthcare coverage through an extended network of public or public–private–partnership hospitals and primary healthcare centers with some features relevant to this study (Martin-Moreno et al., 2009): health care was free of charge except for drugs in non-retired people who had a 40% copayment with a cap on chronic medications; hospital and primary care was financed by the regional governments’ budgets, and healtcare services were organized into HAs, geographically delimited territories with about 100,000–250,000 people served by one public hospital and several primary care centers providing inpatient and outpatient primary and specialized care to the population in its demarcation. Due to these organizational characteristics (geographical planning and minimal accessibility barriers), patients receive most of their care, except highly specialized care, in the HAs they belong to.

### Therapeutic Groups

We selected all drugs (except zoledronate) marketed in 2009 in Spain for osteoporosis treatment categorized into five Anatomical Therapeutic Chemical (ATC) Classification groups: M05BA and M05BB (biphosphonate and combinations, including alendronate, risedronate, ibandronate, etidronate, and alendronate–colecalciferol), M05BX (strontium ranelate), G03XC (raloxifene), H05AA (parathyroid hormone and teriparatide), and H05BA (calcitonin salmon synthetic and elcatonin). Zoledronate was not included because in Spain it is administered on an inpatient basis and there is no register of its use in the outpatient prescription data files.

### Data Sources

Utilization and expenditure data were supplied by the Regional Health Departments from their outpatient drug dispensation data files, containing information on the pharmacy claims reimbursed by the regional healthcare services in 2009. This database includes a description of the drug dispensed (commercial brand, generic name, strength, size of the package, retail price), the patient’s coverage (full or 40% copayment), and the HA in which the prescription was made. The databases transferred to the research team did not contain any information on individual patients. Figures about population entitlement in each HA were obtained from the Health Departments’ entitlement registries.

### Main Measure

This was the defined daily dose (DDD) per 1000 inhabitants and day (DDD/1000/Day) dispensed in each HA according to the pharmaceutical claims reimbursed by the regional governments. The DDD is a technical unit of measurement used in drug utilization studies and assigned by the WHO Collaborating Center for Drug Statistics Methodology (WHO, 2012), equivalent to the average daily maintenance dose of a drug in its main indication for adults. The DDD/1000/Day [(total milligrams consumption in a population aggregated in DDD × 1000)/(365 days × number of inhabitants in the population)] is a rough estimate of the number of people per 1000 inhabitants who are consuming a specific drug at any given moment (point-prevalence). Due to the strong sex-age-related use of osteoporotic drugs (according to Health Departments non-published data, men only account for 5% of prescriptions, and for women only 5% goes to those under 50 years old), we assigned all prescriptions to the population of women aged 50 years and over for simplicity and is thus specified as DDD/1000*W50*/Day from here on.

### Other Measures

(1) The pharmaceutical expenditure on osteoporotic drugs at retail price per women aged 50 years and over and per year; (2) the age–copayment *Indirect Standardized Drug Utilization Rate* (ISR), obtained by comparing the observed DDD/1000W50/Day dispensed in each HA with an estimate of the expected DDD/1000W50/Day in the same HA if its actual population had the same stratified age and copayment drug utilization registered in a given reference population (the region of Madrid).

### Ethical Aspects

The study, observational in design, uses retrospective anonymized non-identifiable and non-traceable data transferred to the research team meeting the requirements of the institutional providers. It is also in accordance with the CIOMS-WHO International Ethical Guidelines for Epidemiological Studies (CIOMS-WHO, 2008) and the Spanish personal data protection and patients rights’ laws (Ley 15/1999, 1999; Ley 41/2002, 2002), and did not require Ethics Committee approval.

### Analysis

First, we estimated the DDD/1000W50/Day by HA. Second, expenditure in each therapeutic class was aggregated by HA and divided by the number of women aged 50 years and over and by DDD dispensed to obtain the annual osteoporotic drug cost per woman aged 50 years and older. Subsequently, *Indirect Standardized Drug Utilization Rates* in each HA were estimated by each therapeutic class as $ISR=observed/\exp ected=\Sigma_{i}{DDD}_{i}/\Sigma_{i}{DUR}_{i}^{R}N_{i}$, where ${DUR}_{i}^{R}$ is the drug utilization rate in the *i*th age–copayment group in a reference population in which these parameters were known. We used the available specific rates from the Madrid Region but recalibrated them to the observed total consumption of the 168 HAs. The indirect method used does not allow the comparison of one HA with another, but does admit a comparison with the reference pattern that by construction is equal to the mean utilization rate of the sNHS for the respective drug. Therefore, an ISR such as 1.50 for a given HA is interpreted as meaning that this HA has a 50% higher consumption of the respective drug than the sNHS average.

A descriptive analysis of drug use (crude and standardized) and spending per woman was carried out presenting the mean, median, and the values of the HAs in percentiles (P) 5, 25, 75, and 95. Variability among HAs was analyzed using the usual SAVA statistics (Ibáñez et al., 2009) but, in order to reduce the influence of those (few) areas with extreme values, we excluded (unless otherwise noted) the HAs outside the 5th and 95th percentile of the corresponding distribution. The statistics used include the high–low ratio (ratio between HAs in the P5 and P95, also known as the extremal quotient; EQ5-95), the interquartile range (ratio between HAs in the P25 and P75; IQR25-75), the coefficient of variation (CoV5-95), the weighted coefficient of variation (CoVW5-95), similar to the CoV5-95 but weighted for the size of the population in each area. The ISRs in each HA and therapeutic class were also displayed as geographical maps (Supplementary Figure S1). Finally, Spearman’s correlation was used to analyze associations among the ISR of the different therapeutic groups; a one-way ANOVA random-effects analysis using the ACs as a factor was used to estimate the proportion of between-HA variance explained by the membership of the HA to one or another region. All the analyses were performed using the STATA 13.0 (StataCorp, College Station, TX, United States) statistical software and R (Free Software Foundation’s GNU General Public License, Boston, MA, United States).

## Results

The average osteoporotic drug consumption in Spain during 2009 was of 116.8 DDD/1000W50/Day, ranging from 78.5 to 158.7 DDD/1000W50/Day between the HAs in the percentiles 5 and 95 (**Table 1**). Seventy-nine percent of the antiosteoporotics consumed were bisphosphonates (91.9 DDD/1000W50/Day) followed by raloxifene (11.2 DDD/1000W50/Day), strontium ranelate (7.1 DDD/1000W50/Day), calcitonins (5.0 DDD/1000W50/Day), and parathyroid hormones including teriparatide (1.6 DDD/1000W50/Day). Regarding variability by therapeutic groups, biphosphonates showed the lowest variation (twice as high in the HA in the 95^th^ percentile compared to that in the 5^th^ percentile) while calcitonins and parathyroid hormones showed the highest variation (10-fold and 18-fold higher consumption in the HA on the P95 compared to the P5, respectively). The standardization by age and copayment did not substantially modify the variability between HAs (Supplementary Table S1).

**Table 1:** Defined daily dose per 1000 women aged 50 years and older and day in osteoporosis drugs by Health Areas (HAs) (Spain, 2009).

	Biphosph.	Strontium R	Raloxifene	Parathyr. H	Calciton.	All
Mean	91.93	7.05	11.20	1.63	5.00	116.80
*SD*	19.09	2.96	5.08	1.28	5.62	24.73
P5	59.85	3.01	3.69	0.24	1.12	78.49
P25	80.27	4.85	7.50	0.69	2.10	99.54
Median	91.59	6.57	10.74	1.32	3.58	116.42
P75	101.10	8.77	14.02	2.19	6.30	130.44
P95	123.28	12.69	20.25	4.22	11.28	158.72
EQ5-95	2.06	4.22	5.49	17.84	10.06	2.02
IQR25-75	1.26	1.81	1.87	3.17	2.99	1.31
CV5-95	0.16	0.33	0.36	0.62	0.60	0.16
CVW5-95	0.15	0.31	0.33	0.56	0.65	0.16


**n* = 168 HAs. SD, standard deviation; P, percentile; EQ, extremal quotient; IQR, interquartile range; CV, coefficient of variation; CWW, weighted coefficient of variation.*

**Table 2** shows the annual treatment expenditure on osteoporosis drugs, which was €426.5 million in the 13 regions, translating into an expenditure of €59.2 for each woman aged 50 years and over, and varying between €38.1 and €83.3 between HAs in the percentiles 5 and 95 (2.2 times higher in the HA on the P95 than in the HA on the P5). Biphosphonates, despite accounting for 78.7% of utilization, only represented 62.9% of total expenditure on osteoporosis drugs, while parathyroid hormones with only 1.6% of utilization accounted for 14.9% of the osteoporosis pharmaceutical spending. Biphosphonates showed the lowest variation in expenditure (2.1 times higher between HAs in the 95 and 5 percentiles), followed by raloxifene (EQ5-95: 4.2) and strontium ranelate (EQ5-95: 5.4), while calcitonin (EQ5-95: 8.4) and parathyroid hormones (EQ5-95: 15.3) showed the highest variation. As with the utilization rates, the standardization by age and copayment did not substantially modify the variability in expenditure per woman between HAs (Supplementary Table S2).

**Table 2:** Osteoporosis drugs expenditure per woman aged 50 years and older by HAs (Spain, 2009).

	Biphosph.	Strontium R	Raloxifene	Parathyr. H	Calciton.	All
Mean	37.23	4.52	5.04	8.84	3.58	59.20
*SD*	7.73	1.90	2.26	6.18	2.61	14.37
P5	24.68	1.94	1.68	1.32	0.97	38.09
P25	32.74	3.12	3.38	4.28	1.89	49.54
Median	36.83	4.21	4.84	7.92	3.03	57.87
P75	41.57	5.65	6.29	11.74	4.53	67.78
P95	52.47	8.17	9.08	20.10	8.13	83.29
EQ5-95	2.13	4.22	5.40	15.27	8.37	2.19
IQR25-75	1.27	1.81	1.86	2.74	2.39	1.37
CV5-95	0.16	0.33	0.36	0.55	0.50	0.19
CVW5-95	0.14	0.30	0.33	0.49	0.51	0.19


*SD, standard deviation; P, percentile; EQ, extremal quotient; IQR, interquartile range; CV, coefficient of variation; CWW, weighted coefficient of variation.*

Spearman correlations between the consumption of the different therapeutic groups (**Table 3**) were, in general, moderate but positive and significant. Three pairs (strontium ranelate with raloxifene and parathyroid hormones, and raloxifene with parathyroid hormones) showed coefficients above 0.40. The ACs explained a substantial part (14%) of the variance in osteoporosis drug utilization rates (**Table 4**), with differences between therapeutic classes, ranging between 15% in the case of biphosphonates and 54% in the case of parathyroid hormones. Supplementary Figure S1 shows the geographical distribution of the indirect standardized rates and the consumption of each therapeutic group by HAs grouped by ACs is shown in Supplementary Figure S2.

**Table 3:** Spearman correlations between HAs drug utilization rates of osteoporosis drugs (Spain, 2009).

	Biphosphonates	Strontium ranelate	Raloxifene	Parathyroid hormones	Calcitonins
Biphosphonates					
Strontium ranelate	0.28				
Raloxifene	0.26	0.46			
Parathyroid hormones	0.22	0.41	0.41		
Calcitonins	0.35	0.34	0.33	0.25	


*All the coefficientes were significant at *p* < 0.05.*

**Table 4:** Variance explained by the Autonomous Community membership of each HA.

	Biphosphonates	Strontium ranelate	Raloxifene	Parathyroid hormones	Calcitonins	All
**Indirect standardized drug utilization rates**						
*r*^2^ (ANOVA)	0.19	0.31	0.40	0.55	0.27	0.19
*p* (ANOVA)	<0.001	<0.001	<0.001	<0.001	<0.001	<0.001
ICC	0.15	0.28	0.39	0.54	0.24	0.14
**Expenditure per woman aged 50 years and older**						
*r*^2^ (ANOVA)	0.19	0.31	0.43	0.44	0.27	0.11
*p* (ANOVA)	<0.001	<0.001	<0.001	<0.001	<0.001	<0.001
ICC	0.15	0.28	0.42	0.43	0.23	0.05


*Random effects ANOVA one-way analysis (Spain, 2009). ICC, intraclass correlation coefficient.*

## Discussion

This population-based study assessed variations in pharmaceutical utilization and spending in osteoporotic drugs between HAs in Spain. Our results provide evidence of an important variability between HAs in the use of antiosteoporotic drugs, with ratios at the 95th percentile more than doubling those at the 5th percentile for all osteoporosis drugs, and reaching an 18-fold difference for some costly drugs such as parathyroid hormones. The extent of these differences between territories was hardly modified by standardizing for age and copayment. Regarding treatment expenditure, we found that variation in drug utilization was transferred almost entirely to variation in expenditure per capita (in this case, per woman aged 50 years and over) and rates of prescribing explained almost all of the variance in drug spending. Furthermore, despite finding a moderate correlation between the use of the different therapeutic groups, our study also shows that antiosteoporotic drugs are prescribed as complementary treatments rather than as a substitute: HA, in general, uses all the groups of antiosteoporotics rather than choosing some groups over others.

Regarding the variability between HA in the use of antiosteoporotic drugs, we found ratios at the 95th percentile more than doubling those at the 5th percentile for all antiosteoporotic drugs altogether, and ranging between a twofold difference for biphosphonates and an 18-fold difference for some costly drugs such as parathyroid hormones. These figures are similar to those reported in previous studies in Portugal (Rocha et al., 2006) and Norway (Devold et al., 2010) for biphosphonates, but much higher for other therapeutic groups as compared with the Portuguese study, which is the only study assesing differences in several antiosteoporotic drug groups. In such study, the amplitude of variation ranged from two to three times across regions for the different antiosteoporotic drugs. Regarding variability for all antiosteoporotic drugs together, the differences found in our study were lower than the differences reported in Canada (Crilly et al., 2014), although the measurements of variability for the latter study are not comparable to those of our study. It might be possible that the higher utilization rates of antiosteporotic drugs evidenced in Spain compared to other European countries (Richards, 2010) translates into higher variability in prescription. The extent of differences in antiosteoporotic drug utilization between territories was hardly modified by standardizing for age and copayment, suggesting a limited role for morbidity (age is a good proxy for the prevalence of osteoporosis) or economic accessibility as a source of variations between geographical territories. This finding should be interpreted from the perspective of ecological studies, avoiding extrapolations to studies on an individual basis in which both of these factors (age and copayment) predict drug consumption accurately. Furthermore, the differential priorization of non-pharmacological interventions between regions (such as promotion of changes in diet, weight bearing exercise, smoking cessation, etc.), together with the variability in the recommendations among clinical pratice guidelines, may have influenced the variability in utilization, but it is unlikely that they do so to the extent of explaining the high variations observed in our study.

We also found that variation in drug utilization is transferred almost entirely to variation in expenditure per capita (in this case, per woman aged 50 years and older) and rates of prescribing explained almost all of the variance in drug spending. This finding suggests that the different amounts of medications prescribed in each territory, rather than their price, are the main source of variation in osteoporosis drug spending in the sNHS setting, in contrast to a large US study using Medicare Part D data, concluding that regional variation in pharmaceutical spending results largely from differences in the costs of drugs rather than prescription volume (Donohue et al., 2012). The discrepancy in findings could be explained by the homogeneity of prices of the most widely used anti-osteoporotic drugs in Spain, derived from a price regulation system that includes administrative approval for the price of brand-name products and a price reference system for drugs with expired patents. This might not be the case for other drug groups with a greater variety in prices or with increased consumption of high-cost groups, or other countries with different price regulatory systems. Our findings suggest that the role of prescription volume and price on pharmaceutical expenditure depends on the drug group and individual context and should be assessed accordingly, avoiding generalization of the results from specific settings.

Lastly, and although the correlation between the use of the different therapeutic groups is moderate, our study also shows that antiosteoporotic medications were used as complementary treatments rather than as a substitute: HAs, in general, use all the groups of antiosteoporotics rather than choosing some groups over others. This finding does not have a clear clinical rationale in osteoporosis, where, in contrast to other conditions such as hypertension, it is exceptional that two different drugs are used simultaneously in the same patient. This tends to amplify variations in global spending and has important implications for the design of practical policies aiming to reduce variability and pharmaceutical expenditure (Zhang et al., 2011).

The evidence accumulated shows wide geographical variation in healthcare utilization and spending, which is independent of patient characteristics and unrelated to the quality of care or patient outcomes (Bernal-Delgado et al., 2009; Mercuri and Gafni, 2011). The results of our study on utilization and spending regarding antiosteoporotic medication are consistent with limited previous research. These results might be indicators of inefficient resource use, and at the same time provide an opportunity for improvement in the quality and value of health care (Gauld et al., 2011; Mays, 2011; Bernal-Delgado et al., 2013). In the specific case of antiosteoporotic medications, an inadequate use (and therefore spending) has been documented in various settings with prescriptions to women with a low risk of fracture and a considerable underuse in people with a high risk of fracture, including those who have already suffered a major osteoporotic fracture (Sanfélix-Genovés et al., 2013b; Wang et al., 2013; Yu, 2017), suggesting the existence of considerable room for improvement in the prevention of osteoporotic fracture.

Our findings are subject to several limitations. First, the nature of the design (ecological) limits the generalization of the results from the geographically aggregated population to the individual basis. Second, the use of data on dispensed drugs may provide an imprecise estimate of a physician’s prescriptions (as some prescriptions issued might not be filled). Third, indirect adjustment for age and copayment may be insufficient to control for differences in the risk of being treated with antiosteoporotic drugs. Fourth, we assigned all DDDs consumed to women aged 50 years and over. Although variations between HAs in utilization in men or young women could affect our estimates of variability, we expect this strategy to have a minor impact on our figures, because the group of women aged 50 years and over is the population group that consumes the vast majority of anti-osteoporotic drugs. Fifth, the results presented correspond to 2009 and might not be extrapolated to the current situation of antiosteoporotic drug use and spending in Spain (which may have been affected by several factors, including safety warnings about bisphosphonates, the expiration of some patents, restrictions of use and/or withdrawal of some drugs, the incorporation of new drugs, as well as policies developed in response to the economic crisis or the impoverishment of the population itself). A very recent study (Martín-Merino et al., 2017) shows trends in the use of antiosteoporotics until 2013, reporting an important decrease during the economic crisis (as it happened with medications aimed to treat other diseases). Also, as some patents have expired from 2009 and new drugs have entered into the market, it is likely that the expenditure in antiosteoporotic drugs has changed in the current context.

Although the level and patterns of use and expenditure might have changed, to our knowledge no other study has assessed the variability in anti-osteoporotic drug prescribing and spending in a comprehensive manner to date, across the Spanish territory, nor in other settings. Therefore, we are presenting novel results, with the most recent information available at the Health Area level regarding this relevant topic.

## Conclusion

In summary, this study highlights a marked geographical variation in the prescription of antiosteoporotic treatments in a setting with universal healthcare coverage, the variation being more pronounced in the case of costly (but low-prescription) drugs such as parathyroid hormones. The differences in rates of prescribing explained almost all of the variance in drug spending, suggesting that the difference in prescription volume between territories, and not the price of the drugs, is the main source of variation in this setting. Our study also shows that antiosteoporotic drugs are prescribed as complementary treatments rather than as a substitute, overall: HAs prescribe all the groups of antiosteoporotics rather than choosing some groups over others. Our findings are useful to identify inconsistencies in the clinical management of a major health problem for older people (especially women) as is osteoporosis, and the prevention of its more severe clinical manifestation: hip fracture. Data on geographical variation of prescription can help guide policy proposals for targeting areas with inadequate antiosteoporotic drug use.

## Member of the Drug Utilization in the Spanish National Health System Research Group

**Aragon:** M^a^ Concepción Celaya, Mercedes Aza, Félix Pradas, Enrique Bernal-Delgado, Anselmo López Cabañas, and María Elfau Maizal; **Andalucia:** Carmen Beltrán Calvo, Teresa Molina López, Carmen Suárez Alemán, Carmen Vela, Lola Pérez Pacheco, M^a^ Ángeles García Lirola, and Francisco Rivas Ruiz; **Asturias:** Gracia Modroño Riaño; **Balearic Islands:** María Vega Martín Martín and Eusebi Castaño; **Canary Islands:** M^a^ Dolores Fiuza Pérez, Jose Luis Alonso Bilbao, Jose Antonio Díaz Berenguer, Alberto Espiñeira Francés, Antonio Cabeza, and Conrado Dominguez Trujillo; **Castilla y León:** Nieves Martín and Judit Ceruelo; **Catalonia:** David Magem; **Extremadura:** Galo Agustín Sánchez Robles, Lola Sainz, Félix Fernández, and Gregorio Montes; **Madrid:** Encarna Cruz Martos and Ángel Mataix; **Murcia:** Rafael Herrero Delicado; **Navarra:** Maite Artázcoz Sanz, Lourdes Muruzábal Sitges, Ana Azparrén Andía, Francisco Javier Garjón Parra, Javier GorrichoMendivil, and Juan Erviti López; **Basque Country:** Felipe Aizpuru Barandiarán, Amanda López Picado, Elena Ruiz de Velasco Artaza, JosuneIribar, and María Armendáriz Cuñado; **Valencia Region:** Gabriel Sanfélix-Gimeno, Salvador Peiró Julián Librero, and María García-Gil.

## Author Contributions

GS-G had full access to all of the data in the study and took responsibility for the integrity of the data and the accuracy of the data analysis. GS-G and SP were responsible for the study concept and design. GS-G and JL-L carried out the data preparation and the statistical analysis. SP, GS-G, and CR-B drafted the manuscript. GS-G, SP, GM-R, JL-L, and CR-B participated in the analysis and interpretation of data, critical revision of the manuscript for important intellectual content, and all approved the final version submitted for publication.

## Role of the Funding

Most of the members of the Drug Utilization in the Spanish National Health System Research Group, work in institutions which depend from the Health Departments of the different Autonomous Communities. These departments or the institutions participating in this research, including the Instituto de Salud Carlos III (which provided the grant for this study), did not play any role in the design of the study; the collection, analysis, or interpretation of data; the writing of the manuscript; or the decision to submit it for publication, and may not necessarily share the contents of this paper.

## Conflict of Interest Statement

SP has received research grants from various pharmaceutical companies, and fees for participation in scientific meetings sponsored by pharmaceutical companies. GS-G participated in 2014 in an advisory meeting of Boehringer Ingelheim. The other authors declare that the research was conducted in the absence of any commercial or financial relationships that could be construed as a potential conflict of interest.
