# Supplementary material for: Variation in Antiosteoporotic Drug Prescribing and Spending Across Spain. A Population-Based Ecological Cross-Sectional Study
Source: Front Pharmacol. 2018 Apr 13;9:342. doi: 10.3389/fphar.2018.00342 (PMC5909183; doi:10.3389/fphar.2018.00342)
Supplement: Supplementary file 1 [file Table_1.doc]

**Supplementary material**

**Variation in antiosteoporotic drug prescribing and spending across Spain. A population-based ecological cross-sectional study.**

**Authors**

**Gabriel Sanfélix-Gimeno, Julián Librero-López, Gracia Modroño-Riaño, Salvador Peiró, Clara L. Rodríguez-Bernal on behalf the Drug Utilization in the Spanish National Health System Research Group.**

| Table S1. Indirect standardized drug utilization rates of antiosteoporotic drugs in women aged 50 and over by Health Areas (Spain, 2009). | | | | | | |
| --- | --- | --- | --- | --- | --- | --- |
|  | Biphosph. | Strontium R | Raloxifene | Parathyr. H | Calciton. | All |
| Min | 0.53 | 0.20 | 0.00 | 0.01 | 0.13 | 0.59 |
| P5 | 0.70 | 0.43 | 0.33 | 0.12 | 0.25 | 0.69 |
| P25 | 0.89 | 0.70 | 0.65 | 0.39 | 0.47 | 0.87 |
| Median | 1.00 | 0.94 | 0.94 | 0.76 | 0.80 | 1.02 |
| P75 | 1.13 | 1.27 | 1.25 | 1.29 | 1.49 | 1.15 |
| P95 | 1.49 | 2.09 | 1.80 | 2.53 | 3.05 | 1.47 |
| Max | 2.02 | 2.64 | 3.10 | 4.77 | 13.64 | 2.07 |
| EQ5-95 | 2.13 | 4.86 | 5.45 | 21.08 | 12.20 | 2.13 |
| IQR25-75 | 1.27 | 1.81 | 1.92 | 3.31 | 3.17 | 1.32 |
| n=168 Health Areas; Min: Minimum, P: Percentile; EQ: Extremal Quotient; IQR: Interquartile Range; Max: maximum; The subindexes indicate that the corresponding statistic was calculated using those mentioned percentiles. | | | | | | |

| Table S2. Indirect standardized expenditure in antiosteoporotic drugs in women aged 50 and over by Health Areas (Spain, 2009). | | | | | | |
| --- | --- | --- | --- | --- | --- | --- |
|  | Biphosph. | Strontium R | Raloxifene | Parathyr. H | Calciton. | All |
| Min | 0.52 | 0.20 | 0.00 | 0.06 | 0.17 | 0.50 |
| P5 | 0.69 | 0.40 | 0.33 | 0.15 | 0.29 | 0.66 |
| P25 | 0.90 | 0.71 | 0.65 | 0.44 | 0.58 | 0.83 |
| Median | 1.00 | 0.94 | 0.93 | 0.87 | 0.96 | 0.97 |
| P75 | 1.13 | 1.26 | 1.26 | 1.27 | 1.39 | 1.15 |
| P95 | 1.52 | 2.10 | 1.81 | 2.38 | 3.07 | 1.62 |
| Max | 2.50 | 2.65 | 3.11 | 4.61 | 7.39 | 2.50 |
| EQ5-95 | 2.20 | 5.25 | 5.48 | 15.87 | 10.59 | 2.45 |
| IQR25-75 | 1.26 | 1.77 | 1.94 | 2.89 | 2.40 | 1.39 |
| n=168 Health Areas; Min: Minimum, P: Percentile; EQ: Extremal Quotient; IQR: Interquartile Range; Max: maximum; The subindexes indicate that the corresponding statistic was calculated using those mentioned percentiles. | | | | | | |

| Figure S1. Maps of indirect Standardized Drug Utilization Rates for osteoporosis medication between Health Areas in Spain, 2009. | |
| --- | --- |
| Biphosphonates | Strontium ranelate |
|  |  |
| Raloxifene | Parathyroid Hormone and teriparatide |
|  |  |
| Calcitonines | Total |
|  |  |
|  | |

| Figure S2. Defined daily dose per 1000 women aged 50 and over, and day, in osteoporosis drugs by Health Areas grouped by Automous Communities (Spain, 2009) | |
| --- | --- |
| Biphosphonates | Strontium ranelate |
|  |  |
| Raloxifene | Parathyroid Hormone and teriparatide |
|  |  |
| Calcitonines | Total |
|  |  |
| The black dots depict the mean DDD/1000W50/Day in each Autonomous Community (identified by the same number in all figures). The burbles depict DDD/1000W50/Day in each Health Area. The bubble size was weighted by the number of women aged 50 and over in each health area. | |
